# Development and Internal Validation of the CLIMPOUT Score: A Multimodal Prediction Model for Neurological Outcome After Pediatric Intracerebral Hemorrhage

**DOI:** 10.3390/diagnostics16172763

**Published:** 2026-08-28

**Authors:** Enrique Pazos, Sara Bobillo-Perez, María Farras-Riu, Francisco Jose Cambra-Lasaosa, Lluïsa Hernandez-Platero, Jordi Muchart, Jùlia Romagosa-Pérez, Christian Stephan-Otto, Iolanda Jordan, Mònica Balaguer

**Affiliations:** 1 Pediatric Intensive Care Unit, Hospital Sant Joan de Deu, Universitat de Barcelona (UB), Passeig Sant Joan de Deu, 2, 08950 Barcelona, Spain; enrique.pazos@sjd.es (E.P.);; 2 Institut de Recerca Sant Joan de Déu (IRSJD), c. de Sta. Rosa, 39, 08950 Barcelona, Spain; 3 Escola de Doctorat Universitat de Barcelona (EDUB), c. Casanova, 143, 08036 Barcelona, Spain; 4 Faculty of Medicine, Universitat de Barcelona (UB), c. Casanova, 143, 08036 Barcelona, Spain; 5 Radiology and Diagnostic Imaging Unit, Hospital Sant Joan de Deu, Universitat de Barcelona (UB), Passeig Sant Joan de Deu, 2, 08950 Barcelona, Spain; 6 Pediatric Computational Imaging Group (PeCIC), Institut de Recerca Sant Joan de Déu (IRSJD), 08950 Esplugues de Llobregat, Spain; 7 Consorcio de Investigación Biomédica en Red de Salud Mental (CIBERSAM), 28029 Madrid, Spain; 8 Faculty of Medicine, University of Vic-Central University of Catalonia (UVic-UCC), 08500 Vic, Spain

**Keywords:** pediatric sICH, outcome prediction, multimodal predictive model, quantitative imaging, functional outcome prediction, automated volumetry

## Abstract

Spontaneous pediatric intracerebral hemorrhage (sICH) is a rare but devastating neurological emergency associated with high mortality and long-term disability. Existing prognostic scores rely on a reduced number of variables and have shown limited validation, highlighting the need for comprehensive tools. This study describes the protocol for the development and internal validation of the CLIMPOUT (Clinical, Laboratory, and Imaging Predictive Outcome) score, a multimodal model designed to improve early prognostic assessment in pediatric sICH. This single-center retrospective cohort study will include consecutive patients (1 month–18 years) admitted to a tertiary pediatric intensive care unit (PICU) with spontaneous sICH between 2018 and 2025. Predictor variables will be restricted to baseline information available at PICU admission, including demographics, clinical status, laboratory markers, and quantitative neuroimaging features. Hematoma and intraventricular hemorrhage volumes will be measured using standardized three-dimensional segmentation. The primary outcome will be poor neurological status at 12 months, defined by the Pediatric Stroke Outcome Measure (PSOM). We will develop a multivariable logistic regression model following contemporary recommendations, including prespecified predictor selection, multiple imputation for missing data, and internal validation by 1000 bootstrap resamples. The CLIMPOUT score is expected to identify independent predictors of poor neurological outcome and provide a standardized, multimodal risk stratification model with improved prognostic performance compared with currently available pediatric scores. This protocol establishes a rigorous framework for developing a clinically applicable prediction model that may support early individualized decision-making, optimize neurocritical care management, and provide the foundation for future multicenter validation.

## 1. Introduction

Spontaneous pediatric intracerebral hemorrhage (sICH) represents a significant clinical and public health challenge, accounting for up to 54% of all childhood strokes, a proportion significantly higher than observed in the adult population [1]. In developed countries, pediatric sICH represents a leading cause of long-term neurodevelopmental morbidity, severe functional disability, and mortality [1,2,3]. A critical distinction lies in the underlying etiology; while adult cases are dominated by hypertensive hemorrhage or cerebral amyloid angiopathy, pediatric sICH is typically linked to structural lesions, non-traumatic vascular ruptures, and congenital vascular malformations, requiring distinct and immediate neurocritical diagnostic approaches [4]. In current clinical practice, the acute management of pediatric hemorrhagic events relies on a comprehensive and timely assessment of clinical status, vital signs, laboratory parameters, and neuroimaging findings [1,5].

However, a significant and persistent gap exists between adult and pediatric diagnostic frameworks. In adults, well-established predictive models, such as the classic adult sICH score, utilize variables like the Glasgow Coma Scale (GCS), age, and radiological markers to accurately estimate mortality and functional prognosis [6,7]. In contrast, prognostic factors in the pediatric population remain insufficiently defined and lack robust validation across diverse patient cohorts [4,8]. A major point of divergence in the literature is the suboptimal applicability of adult-based predictive tools to children. It is increasingly recognized that pediatric neurological recovery follows a distinct biological trajectory due to age-dependent brain plasticity, evolving skull compliance, and different physiological compensatory mechanisms, making adult models poor predictors for younger patients [5]. Despite its clinical impact, pediatric sICH remains understudied, with a lack of dedicated evidence-based management and prognostic guidelines. Recently, the first international multidisciplinary consensus has highlighted the critical need for structured, expert-driven guidance to reduce practice variability in the acute management of these patients [3].

While specific factors have been identified in children—such as clinical severity indices and acute secondary complications [2,6,8]—the most critical radiological predictor remains the total intracerebral hemorrhage volume relative to total brain volume (TBV) [9,10]. Historically, existing risk-stratification models rely on the classic ABC/XYZ geometrical formula to estimate bleeding size, which represents an adaptation of the adult ABC/2 method to pediatric head CT dimensions [10,11]. Adapting purely ellipsoidal geometric formulas to pediatric populations presents clear limitations; spontaneous hemorrhages in children frequently exhibit highly irregular, multi-lobular, or non-spherical morphologies. Consequently, the traditional ABC approach struggles to accurately capture these complex structures, occasionally leading to severe volume underestimation or unacceptable inter-observer variability [9,10].

In an attempt to standardize these metrics, Beslow et al. [12] used this geometric approach to propose the pediatric sICH score, which relies exclusively on radiological parameters. Similarly, Guédon et al. [13] developed a modified pediatric score that incorporates both imaging and initial neurological status. Despite their promise, these existing pediatric tools face significant limitations: while the modified scale by Guédon et al. has achieved successful independent external validation [13], both scales remain predominantly focused on restricted subsets of data, failing to comprehensively integrate acute clinical indicators alongside metabolic laboratory variables, which restricts their global multi-domain predictive accuracy [12,13]. Furthermore, the potential of objective, digital neuroimaging analysis tools to reduce inter-observer variability in this field has yet to be fully explored [9,12]. The development of comprehensive models that objectively synthesize clinical, laboratory, and imaging data represents a key opportunity to optimize the management of pediatric hemorrhagic stroke [3,4,5].

Beyond the initial structural injury, spontaneous pediatric sICH initiates a complex cascade of secondary brain insults. Following the primary bleed, perihematomal neuroinflammation, oxidative stress, blood–brain barrier disruption, and cytotoxic edema occur, significantly contributing to progressive tissue destruction and delayed neurological deterioration. Unlike adult cases driven primarily by chronic hypertension, the pediatric response to acute brain injury follows a distinct physiological trajectory involving unique systemic and metabolic stress patterns [14]. Understanding these secondary metabolic dynamics is crucial, as they underscore the need for multimodal prediction models that incorporate early biochemical markers alongside precise volumetric imaging [14]. Given the lack of standardized prognostic guidelines, recent international consensus emphasizes the urgent need for structured, expert-driven stratification tools to reduce practice variability and optimize care during the acute phase [3].

Therefore, the aim of the present study is to describe the protocol for the development and internal validation of the CLIMPOUT (Clinical, Laboratory, and Imaging Predictive Outcome) Score, a multimodal prediction model designed to estimate poor long-term neurological outcome in children with spontaneous intracerebral hemorrhage admitted to a tertiary pediatric intensive care unit. By integrating standardized clinical variables, laboratory biomarkers, and quantitative neuroimaging features obtained through three-dimensional volumetric analysis, this study aims to establish a reproducible methodological framework for individualized risk prediction and to provide the basis for external validation and clinical implementation.

## 2. Materials and Methods

### 2.1. Study Design and Setting

This is a single-center, retrospective, observational cohort study designed to develop and internally validate a multimodal predictive score. The study will be conducted exclusively at the Pediatric Intensive Care Unit (PICU) of Hospital Sant Joan de Déu (Esplugues de Llobregat, Barcelona, Spain). Our department is a specialized, tertiary-level academic referral center for pediatric neurocritical care, stroke, and complex pathologies, serving a large catchment area both nationally in Spain and across Europe. Conducting this research within a single high-volume center helps minimize variability in neurocritical therapeutic protocols, supportive intensive care management, and imaging acquisition parameters, reducing a common source of confounding in multi-center predictive models for rare pediatric conditions.

### 2.2. Patient Selection and Eligibility

Patients will be identified through a comprehensive retrospective review of electronic health records spanning the period from 2018 to 2025. Patients will be screened for eligibility based on the following criteria: Inclusion Criteria: All pediatric patients (aged 1 month to 18 years) with a confirmed diagnosis of non-traumatic, pediatric sICH via neuroimaging admitted to the PICU will be consecutively enrolled. Exclusion Criteria: (1) Neonates aged 0 to 28 days are explicitly excluded from this cohort due to distinct underlying etiologies, immature cerebral compliance, and completely different physiological recovery trajectories; (2) ICH secondary to head trauma, accidental or not; (3) hemorrhagic transformation of a previous ischemic stroke or cerebral venous sinus thrombosis; (4) patients with a pre-existing moderate-to-severe neurological disability or significant baseline neurodevelopmental delay; (5) lack of baseline neuroimaging within the first 24 h of ictus; (6) ICH secondary to a previously diagnosed brain tumor; and (7) bleeding directly attributable to recent neurosurgical interventions.

### 2.3. Clinical Data

A multidisciplinary team will extract data from electronic health records using a standardized case report form (CRF). To ensure the robustness of the prognostic model, data extraction will be categorized into two distinct groups.

Predictor variableswill be strictly restricted to information available at the time of PICU admission, including demographics, exact age (calculated in decimal years or months), biological sex, weight, height, initial neurological status (admission GCS), baseline laboratory parameters (glucose, lactate, sodium, bicarbonate), and baseline neuroimaging features. Overall systemic illness severity at admission will be quantified using the Pediatric Risk of Mortality III (PRISM III) score.

Descriptive data regarding therapeutic interventions (hyperosmolar therapy, neurosedation, surgical procedures) and secondary complications arising during the PICU stay (e.g., nosocomial infections, acute organ failure, or progress towards brain death) will be systematically collected to characterize the cohort and the quality of care. However, these descriptive variables will be excluded from the final prediction score construction to prevent look-ahead bias and ensure the model’s clinical applicability for early risk assessment.

Furthermore, continuous hemodynamics, multimodal neuromonitoring (ICP, CPP, NIRS, and transcranial Doppler peak values), blood product administration, anticonvulsant regimens, and stroke etiology (hematological, metabolic, infectious, vascular malformations, etc.) will be registered to provide a comprehensive profile of the pediatric sICH cohort.

Although emergency neuroimaging obtained upon presentation serves as the baseline evaluation for model construction, all predictor variables included in the final model—including neuroimaging, clinical and laboratory parameters—are restricted to information available at or before PICU admission, consistent with the admission-based design described above.

### 2.4. Laboratory Data

Blood samples obtained strictly at the time of PICU admission will be processed to evaluate systemic homeostasis and early metabolic or organ breakdown. A comprehensive profile of interest will be extracted from institutional laboratory registries, including full baseline hematological parameters and thorough coagulation profiling, which encompasses thrombin time, prothrombin time, activated partial thromboplastin time (aPTT), and fibrinogen concentration.

Early metabolic failure and cellular stress responses will be primarily quantified using initial stress-induced blood glucose levels, blood lactate concentrations, and core serum sodium levels. Acid-base equilibrium and oxygenation efficiency will be evaluated via immediate arterial blood gas analysis, specifically registering partial pressure of oxygen (pO2), partial pressure of carbon dioxide (pCO2), and overall acid-base balance variables. Within this metabolic diagnostic framework, special predictive emphasis will be placed on absolute bicarbonate levels (mmol/L) and baseline base excess (mEq/L), serving as primary quantitative surrogates for systemic hypoperfusion, metabolic acidosis, and early physiological collapse associated with acute neurological injury.

### 2.5. Radiological Data and Image Analysis

This study introduces an automated radiological approach to obtain quantitative imaging metrics. Initial Digital Imaging and Communications in Medicine (DICOM) images will be processed to determine: (a) anatomical location (supratentorial vs. infratentorial and specific location); and (b) presence of intraventricular hemorrhage (IVH) or hydrocephalus.

#### 2.5.1. Hematoma Volumetry and Ventricular Blood Quantification

**Figure 1:**
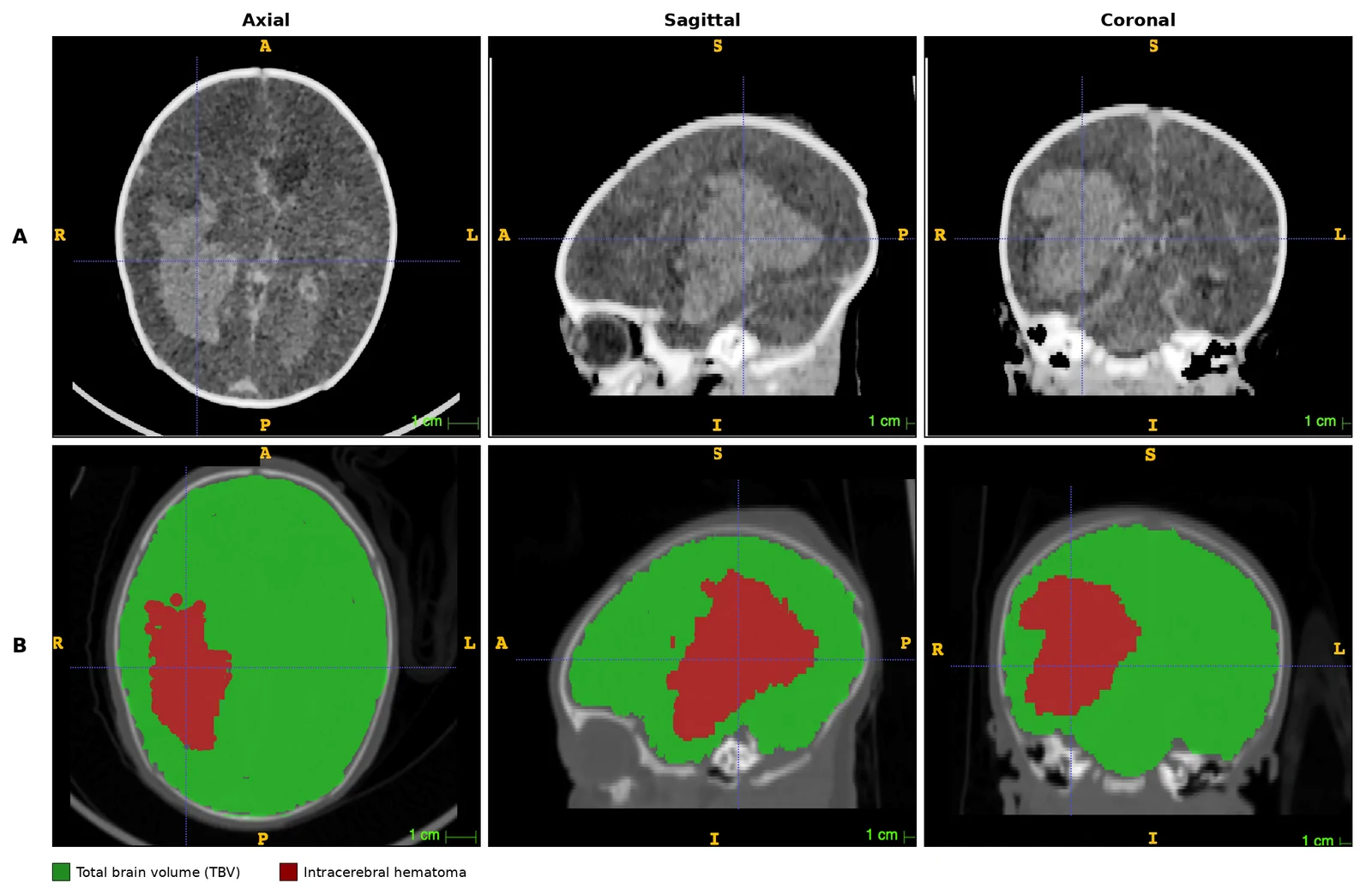
Quantitative neuroimaging analysis using ITK-SNAP. (**A**) Baseline non-contrast head CT in axial, sagittal, and coronal planes, showing a spontaneous pediatric intracerebral hemorrhage with mass effect. (**B**) Corresponding three-dimensional volumetric segmentation, with total brain volume (green) and intracerebral hematoma (red) labeled to compute hematoma-to-brain volume ratios and midline shift. Orientation labels follow standard radiological convention: A, anterior; P, posterior; S, superior; I, inferior; R, right; L, left.

Three-dimensional digital volumetry of the parenchymal hematoma will be performed through semi-automatic, slice-by-slice segmentation of non-contrast head CT scans using the Segment Editor module of 3D Slicer (version 5.8.1) [15], with manual refinement performed in ITK-SNAP software (version 4.0) [16]. Total brain volume will be obtained after skull-stripping using SynthStrip [17], enabling evaluation of the structural lesion alongside the total brain volume (Figure 1). To avoid measurement bias and ensure reproducibility, all volumetric segmentations will be performed independently by two independent investigators blinded to the patients’ clinical course and final outcomes. Inter-observer reliability will be mathematically assessed using the Dice Similarity Coefficient (DSC), setting a minimum acceptable threshold of DSC > 0.85. Patients presenting with intraventricular hemorrhage (IVH) extension will not be excluded from the radiological protocol. Instead, the intraventricular blood volume will be quantified using the same semi-automatic 3D segmentation approach described above.

#### 2.5.2. Structural Mass-Effect and Space-Occupying Parameters

The physical impact of the hematoma will be strictly quantified through three standardized neuroradiological metrics:

1. Midline Shift (MLS): Measured in millimeters at the level of the foramen of Monro, calculating the maximum perpendicular distance from the ideal midline (drawn between the anterior and posterior attachments of the falx cerebri) to the septum pellucidum.
2. Basal Cisterns Effacement: Categorized semi-quantitatively according to a 3-tier scale: Grade 0 (Normal, fully visible cisterns), Grade 1 (Partially compressed/effaced), and Grade 2 (Completely obliterated/absent perimesencephalic and suprasellar cisterns).
3. Acute Secondary Hydrocephalus: Evaluated through the Evans’ Index (the ratio of the maximum width of the frontal horns of the lateral ventricles to the maximum internal diameter of the skull), where a ratio > 0.30 in the presence of downstream ventricular pathway obstruction will define acute obstructive hydrocephalus.

#### 2.5.3. Imaging Windowing and Expansion Analysis

Only baseline head CT scans obtained within the first 24 h from symptom onset will be used for the initial CLIMPOUT score calculation, consistent with the admission-based predictor window defined in Section 2.3.

### 2.6. Outcome Assessment

The primary clinical outcome will be long-term functional neurological status assessed at 12 months post-discharge utilizing the Pediatric Stroke Outcome Measure (PSOM) score [18]. Long-term follow-up and PSOM evaluations are conducted during routine scheduled face-to-face clinical visits by a pediatric neurologist. A poor neurological outcome will be defined a priori as a final PSOM score ≥ 2 at 12 months (indicating moderate to severe neurological deficit or death), while a favorable outcome will be defined as a PSOM score < 2 [19].

### 2.7. Statistical Analysis Plan

Continuous variables will be described using mean (standard deviation, SD) or median (interquartile range, IQR) according to data distribution, while qualitative variables will be expressed as frequencies and percentages.

The protocol follows the Transparent Reporting of a multivariable prediction model for Individual Prognosis Or Diagnosis (TRIPOD) statement for prediction model development. Sample size adequacy was determined based on the recommendations by Riley et al. [20], targeting a shrinkage factor of ≥0.90. To ensure model stability and account for our institutional annual patient volume and historical incidence over the 7-year inclusion period (2018–2025), we have prespecified a candidate set of six key predictors to be evaluated in the regression model based on clinical expertise and previous research (namely: admission Glasgow Coma Scale, blood glucose, serum bicarbonate, relative intracranial hemorrhage volume, intraventricular hemorrhage volume, and midline shift). The sample-size estimation was mathematically modeled based on these six candidate parameters. With an estimated 60 outcome events and six candidate predictors, this yields an events-per-variable (EPV) ratio of approximately 10, considered robust for minimizing the risk of overfitting.

Model development will involve: (1) Predictor selection of prespecified clinical variables, coded appropriately (continuous variables evaluated for linearity using restricted cubic splines, modeled linearly where appropriate, and binary/categorical variables coded using standard indicator dummy variables). (2) Handling of missing data via multiple imputation by chained equations (MICE) under the missing-at-random assumption, generating 20 imputed datasets, with the entire imputation process fully integrated within each bootstrap resampling iteration to properly account for missing-data uncertainty. (3) Control of overfitting through internal validation using 1000 bootstrap resamples to obtain optimism-corrected performance estimates. (4) Conversion of regression coefficients into integer-based points to create a practical bedside scoring system following Sullivan’s methodology. Following this conversion, the discrimination (AUC-ROC) and calibration (intercept, slope, and Brier score) of the simplified integer-based score will be formally reassessed using the same bootstrap framework to ensure that rounding does not compromise predictive performance.

Finally, the incremental predictive value of the CLIMPOUT model over existing pediatric sICH scores (Beslow et al. and Guédon et al.) will be formally assessed by comparing AUC-ROC curves using DeLong’s test, alongside evaluation of calibration plots and decision curve analysis across clinically relevant threshold probabilities. For all statistical tests, significance was set a priori at a two-tailed *p*-value < 0.05.

All statistical analyses will be performed using R software (version 4.2.2) and IBM SPSS Statistics (version 29.0; IBM Corp., Armonk, NY, USA).

### 2.8. Ethical Considerations and Data Collection

The study will be conducted in accordance with the ICH E11 Guideline and the Declaration of Helsinki (Fortaleza, Brazil, 2024 revision). The study protocol has been approved by the Ethics Committee of Sant Joan de Déu Hospital (PIC-169-24). Complete identifying information and consent forms will be securely stored in the investigator’s files. To ensure scientific rigor and data accessibility, the study data will strictly comply with the FAIR criteria (Findable, Accessible, Interoperable, and Reusable). The processing, communication, and transfer of personal data of all participating patients will comply with Regulation (EU) 2016/679 (General Data Protection Regulation) of 27 April 2016 and Organic Law 3/2018, of 5 December, on the Protection of Personal Data and Guarantee of Digital Rights (LOPDPGDD). Accordingly, all collected data will be strictly pseudonymized: each patient will be assigned a unique study number that will prevent their direct or indirect identification. Access will be limited to physicians and clinical researchers directly involved in the study, the Ethics Committee, and relevant health authorities, ensuring strict compliance with regulatory and ethical standards. Furthermore, a comprehensive Data Management Plan (DMP) will be developed to describe and define each data management activity carried out during the study. This document will be prepared by the designated data manager and systematically reviewed and approved by the principal investigator and the statistician to ensure strict compliance with regulatory and ethical standards.

## 3. Limitations

The present study has limitations that should be acknowledged. First, the strictly retrospective nature of the data collection for the 2018–2025 period may introduce inherent historical biases, such as missing or incomplete clinical records in older electronic files. To mitigate this factor, a highly standardized electronic case report form (CRF) has been deployed, and only patients featuring complete baseline imaging and long-term functional primary outcome measures will be included in the final analysis.

Second, because spontaneous hemorrhagic stroke is an exceptionally rare condition in the pediatric population compared to adults, the overall available sample size is naturally constrained. This lower incidence could limit the total number of absolute events available for multivariable validation. To rigorously counteract this limitation without compromising statistical validity, the study exploits the high-volume nature of our institution as a major national and European tertiary referral hub for complex neurocritical pathology, consecutively reviewing data over an extensive 7-year timeframe to maximize patient recruitment. Consequently, this project is framed as a robust exploratory analysis designed to establish the necessary preliminary score metrics. This foundational protocol will lay the rigorous methodological groundwork required to subsequently pilot and launch a prospective, multi-center validation study.

Third, while the digital volumetry relies on specialized software, accuracy is dependent on the initial quality of the CT scans, where movement artifacts or emergency slice thicknesses typical of critical care settings could influence the calculation.

Finally, the functional long-term neurological assessment using the PSOM at 12 months post-discharge mandates a high rate of longitudinal compliance.

## 4. Discussion

Existing pediatric scores have substantially advanced prognostic assessment but remain limited by their reliance on a relatively restricted number of clinical and radiological predictors. The CLIMPOUT study has been designed to address this unmet need by establishing a rigorous methodological framework for the development of a prediction model that combines clinical characteristics, laboratory biomarkers and quantitative neuroimaging variables to estimate long-term neurological outcome.

One of the main strengths of the proposed model lies in its multimodal design. Previous pediatric prognostic scores, including the pediatric sICH score proposed by Beslow et al. [12] and its subsequent modification by Guédon et al. [13], marked important advances in pediatric neurocritical care by demonstrating the prognostic value of neurological examination and radiological severity. However, these models rely on a relatively limited number of predictors and do not incorporate several routinely available physiological and biochemical variables that may reflect the systemic response to acute brain injury. Increasing evidence suggests that neurological outcome after pediatric sICH is determined not only by structural injury but also by secondary brain insults, systemic metabolic disturbances and critical care interventions occurring during the hyperacute phase [1,6]. Integrating these complementary domains may therefore improve prognostic accuracy beyond that achieved by imaging-based scores alone.

Another innovative aspect of the CLIMPOUT protocol is the incorporation of quantitative three-dimensional neuroimaging analysis. Hematoma volume has consistently been identified as one of the strongest predictors of outcome after pediatric sICH [6,9]. Nevertheless, most previous studies estimated hemorrhage size using simplified geometric approaches such as the ABC/XYZ method [10,11]. Although these techniques are practical in emergency settings, they assume an ellipsoid hematoma morphology and may substantially underestimate irregular or multilobulated hemorrhages, which are frequently encountered in children. By implementing standardized three-dimensional segmentation using ITK-SNAP [15], the present protocol aims to obtain more accurate volumetric measurements while reducing interobserver variability through blinded independent assessment and formal evaluation of measurement agreement. This methodology also enables the incorporation of additional quantitative imaging biomarkers, including relative hematoma burden (hematoma volume relative to total brain volume) [9], ventricular extension and objective measures of mass effect, potentially improving the biological representation of disease severity.

Beyond predictor selection, an important contribution of the CLIMPOUT study is its emphasis on methodological quality. The protocol has been designed following contemporary recommendations for prediction model development, incorporating predefined eligibility criteria, standardized data collection, appropriate sample size considerations, multivariable modelling, internal validation through bootstrap resampling and comprehensive evaluation of model performance, including both discrimination and calibration. These methodological elements are increasingly recognized as essential for minimizing optimism, reducing overfitting and facilitating reproducibility and future external validation. In rare pediatric diseases, where multi-center datasets are often difficult to obtain, methodological rigor becomes particularly important to maximize the reliability and transportability of predictive models.

If successfully developed and validated, the CLIMPOUT score could provide clinicians with an objective and standardized tool for early risk stratification in children with spontaneous intracerebral hemorrhage. By integrating routinely available clinical, laboratory, and quantitative imaging variables obtained shortly after admission, the model may support decision-making in the emergency department and pediatric intensive care unit by identifying patients at increased risk of unfavorable neurological outcome. This information could complement, rather than replace, clinical judgment when prioritizing admission to specialized neurocritical care units, tailoring the intensity of neurological monitoring, selecting patients for early neurosurgical evaluation, and guiding timely neuroprotective interventions. Furthermore, individualized prognostic estimates may facilitate multidisciplinary care planning by enabling the early initiation of personalized rehabilitation programs and improving communication with families regarding expected neurological recovery [5,18], as well as optimizing the allocation of critical care resources.

Beyond individual patient care, a standardized and reproducible risk stratification tool could contribute to the harmonization of patient selection in future clinical trials evaluating novel neuroprotective therapies, allowing more homogeneous study populations and facilitating comparisons across centers. Such standardization may ultimately improve the evaluation of emerging neuroprotective interventions and support the development of precision medicine strategies in pediatric neurocritical care.

Future perspectives include the external validation in independent multicenter cohorts to assess the model’s transportability across different healthcare systems and clinical settings. Future iterations may also incorporate advanced neuroimaging techniques, including magnetic resonance imaging biomarkers such as diffusion-weighted imaging, susceptibility-weighted imaging, or quantitative measures of white matter injury, potentially improving predictive performance beyond conventional computed tomography.

In parallel, advances in artificial intelligence and automated image analysis may enable fully automated extraction of quantitative imaging biomarkers, substantially reducing observer dependency and facilitating implementation in routine clinical practice. As larger multicenter datasets become available, the model could also evolve through periodic recalibration and dynamic updating using modern machine learning methodologies, ensuring sustained predictive performance and continuous adaptation to changing clinical practice [21].

In conclusion, the CLIMPOUT protocol proposes a comprehensive methodological framework for developing a multimodal prediction model for pediatric spontaneous intracerebral hemorrhage. By integrating standardized clinical information, laboratory biomarkers and advanced quantitative neuroimaging within a robust statistical framework, this study seeks to overcome several limitations of existing prognostic tools and establish the foundations for future externally validated precision medicine strategies in pediatric neurocritical care. Ultimately, the long-term vision of the CLIMPOUT project is not only to predict outcome, but also to facilitate personalized neurocritical care by supporting timely, evidence-based clinical decisions throughout the continuum of care.

## Data Availability

The original contributions presented in this study are included in the article. Further inquiries can be directed to the corresponding author.
